# AN ASSESSMENT OF STATE EMPLOYEE BACKGROUND CHECK POLICIES FOR IMPROVING QUALITY IN NURSING FACILITIES

**DOI:** 10.1093/geroni/igae098.4308

**Published:** 2024-12-31

**Authors:** Gul Rukh Mehboob, Brian Kaskie, Hari Sharma

**Affiliations:** University of Iowa - Iowa City, IA, Iowa City, Iowa, United States; University of Iowa, Iowa City, Iowa, United States; University of Iowa - Iowa City, IA, Iowa, Iowa, United States

## Abstract

Employee background checks are important measures for ensuring the safety of older adults in nursing homes. Programs like the National Background Check Program (NBCP) provide federal grants to help states enhance their background check systems for long-term care receivers. While these checks require resources, their effectiveness in reducing abuse, neglect, and exploitation remains uncertain. We aim to compare NBCP grant-receiving states (N=27) with non-receiving states (N=23) to identify regulatory changes and adaptation of specific policy measures over time to improve employee background check systems. We used a policy scan approach to compare and rank states by thoroughly analyzing regulations, bills, documents and reports from 2005 to 2024. We evaluated states based on three policy domains: types of LTC providers required to conduct employee background checks, the disqualifying offenses considered by the states, and the processes used to implement and administer these checks. We also analyzed temporal changes in states’ background check systems over time. We found notable differences in regulations concerning the types of LTC providers involved and disqualifying offenses between NBCP and non-NBCP states. However, we found little difference in the processes between these two groups. Overall, the NBCP states show better adaptation and temporal changes due to advanced technical systems and improved regulations. To our knowledge, this is the first study to comprehensively assess the regulatory framework of state employee background check systems. Our study has direct policy implications for state and national policymakers debating the effectiveness of NBCP in improving nursing home quality.

